# Insect Cell-Derived Cofactors Become Fully Functional after Proteinase K and Heat Treatment for High-Fidelity Amplification of Glycosylphosphatidylinositol-Anchored Recombinant Scrapie and BSE Prion Proteins

**DOI:** 10.1371/journal.pone.0082538

**Published:** 2013-12-18

**Authors:** Morikazu Imamura, Nobuko Kato, Hiroyuki Okada, Miyako Yoshioka, Yoshifumi Iwamaru, Yoshihisa Shimizu, Shirou Mohri, Takashi Yokoyama, Yuichi Murayama

**Affiliations:** 1 Influenza • Prion Disease Research Center, National Institute of Animal Health, Tsukuba, Ibaraki, Japan; 2 Research Area of Pathology and Pathophysiology, National Institute of Animal Health, Tsukuba, Ibaraki, Japan; Van Andel Institute, United States of America

## Abstract

The central event in prion infection is the conformational conversion of host-encoded cellular prion protein (PrP^C^) into the pathogenic isoform (PrP^Sc^). Diverse mammalian species possess the cofactors required for *in vitro* replication of PrP^Sc^ by protein-misfolding cyclic amplification (PMCA), but lower organisms, such as bacteria, yeasts, and insects, reportedly lack the essential cofactors. Various cellular components, such as RNA, lipids, and other identified cofactor molecules, are commonly distributed in both eukaryotes and prokaryotes, but the reasons for the absence of cofactor activity in lower organisms remain to be elucidated. Previously, we reported that brain-derived factors were necessary for the *in vitro* replication of glycosylphosphatidylinositol-anchored baculovirus-derived recombinant PrP (Bac-PrP). Here, we demonstrate that following protease digestion and heat treatment, insect cell lysates had the functional cofactor activity required for Bac-PrP replication by PMCA. Mammalian PrP^Sc^ seeds and Bac-PrP^Sc^ generated by PMCA using Bac-PrP and insect cell-derived cofactors showed similar pathogenicity and produced very similar lesions in the brains of inoculated mice. These results suggested that the essential cofactors required for the high-fidelity replication of mammalian PrP^Sc^ were present in the insect cells but that the cofactor activity was masked or inhibited in the native state. We suggest that not only RNA, but also DNA, are the key components of PMCA, although other cellular factors were necessary for the expression of the cofactor activity of nucleic acids. PMCA using only insect cell-derived substances (iPMCA) was highly useful for the ultrasensitive detection of PrP^Sc^ of some prion strains.

## Introduction

Transmissible spongiform encephalopathies (TSEs), including scrapie in sheep, bovine spongiform encephalopathy (BSE) in cattle, and Creutzfeldt-Jakob disease in humans, are infectious and fatal neurodegenerative diseases [1]. Unique proteinaceous infectious agents called prions are considered to be the cause of TSEs. Prions consist primarily of a pathogenic form (PrP^Sc^) of the normal cellular prion protein (PrP^C^) and PrP^Sc^ appears to propagate itself via autocatalytic conformational conversion of PrP^C^ [2]. Although the conversion mechanism of PrP^C^ into PrP^Sc^ remains unclear, host cofactors have been suggested to be necessary for the efficient replication of PrP^Sc^ in addition to the PrP^C^ substrate [3]. Various biological molecules including nucleic acids, sulfate glycans, lipids, proteins [4], [5], [6], [7], [8], [9], [10], and nicotinamide adenine dinucleotide phosphate (NADPH) [11] have been reported to act as cofactors for the conversion of PrP^C^ into PrP^Sc^, suggesting that it is not possible to attribute cofactor activity to a specific molecule. Furthermore, the dependency on such cofactors varies among the animal or prion strains examined [12]. Therefore, a more detailed analysis of the cofactors is needed to identify the conversion mechanism of PrP^C^ and to initiate the development of new potential therapies targeting cofactors.

Protein misfolding cyclic amplification (PMCA) is used to amplify minute amounts of PrP^Sc^ to easily detectable levels and its usefulness for highly sensitive and specific detection of prions has been established [13], [14], [15], [16], [17]. Typically, brain homogenate from normal animals is used as the PrP^C^ substrate for amplification and PrP^Sc^ amplified by PMCA retains similar biochemical and biological characteristics to the prion strain used as the PrP^Sc^ seed [18]. Therefore, PMCA using brain homogenates is expected to mimic the *in vivo* replication of PrP^Sc^ and it is a useful experimental system to elucidate the complexity of prions, such as the existence of prion strains and the PrP^Sc^ replication mechanism. Indeed, PMCA has been used in studies concerning species barriers in prion transmission [19], [20], [21] and to investigate the cofactors involved in the replication of PrP^Sc^ [12], [22].

Abid *et al.* [22] suggested that the cofactors required for amplification of 263K PrP^Sc^ are contained in various mammalian tissues such as the liver, kidney, heart, and muscles of mammals, whereas lower organisms such as bacteria, yeasts, and flies do not possess cofactor activities. Previously, we reported that baculovirus-derived recombinant PrP (Bac-PrP) modified with glycosylphosphatidylinositol (GPI) was converted by PMCA (Bac-PMCA) into a PrP^Sc^ form (Bac-PrP^Sc^) that retained the strain-specific characteristics of the PrP^Sc^ seed used [23]. Bac-PrP^Sc^ was not amplified with insect cell-derived substances alone and the addition of *Prnp^0/0^* mouse brain homogenate to the reaction mixture was necessary for amplification [23]. These observations suggested that mammal-derived cofactors were also essential for *in vitro* Bac-PrP^Sc^ replication. However, the cofactors or cofactor candidates identified to date are universal cellular components common to both eukaryotes and prokaryotes [4], [5], [6], [7], [8], [9], [10]. More specifically, RNA derived from worms, as well as mammalian RNA, efficiently amplified hamster scrapie prions [24]. Furthermore, prion-like proteins, which are transmissible proteins accompanied by a structural conversion of protein, are well known in yeasts [25]. Therefore, it is probable that non-mammalian species also possess cofactors that can induce the post-translational structural conversion of proteins. The apparent absence of cofactor activity in non-mammalian species may be due to its functional status; that is, cofactors of non-mammalian species may be in a non-functional state for the replication of mammalian prion proteins in cells.

Identifying the differences between mammalian and non-mammalian cofactors will contribute much towards our understanding of the mechanism of conformational changes of prion proteins. Reconstitution experiments using recombinant PrP and known materials are needed to achieve this purpose. Although *Escherichia coli*-derived GPI-anchorless recombinant PrP was available as a PrP^C^ substrate in a PMCA reaction, the biological characteristics of the amplified PrP^Sc^ were different from those of the PrP^Sc^ seed [26]. On the other hand, GPI-anchored recombinant Bac-PrP^Sc^ with the same strain-specific characteristics as the PrP^Sc^ seed was generated by PMCA, with similar outcomes to conventional PMCA using brain homogenates. In the current study, we therefore reevaluated the cofactor activity for high-fidelity replication of PrP^Sc^ using the Bac-PMCA system.

## Materials and Methods

### Ethics Statement

The animal experiments were approved by the Committee of Animal Experiment in National Institute of Animal Health (approval ID: 11-008, 13-005) and were performed in accordance with the Guideline for Animal Experiment at the Ministry of Agriculture, Forestry, and Fisheries of Japan.

### Partial purification of Bac-PrP and brain-derived PrP^C^ by immobilized metal ion affinity chromatography

Insect cells expressing Bac-PrP (1×10^8^ cells) were lysed in 20 ml of TALON™ xTractor buffer (Clontech, Mountain View, CA) containing EDTA-free protease inhibitor cocktail (Nacalai Tesque, Kyoto, Japan) on ice with occasional agitation for 15 min. Following incubation, the cell lysate was centrifuged at 20,000× *g* for 20 min at 4°C and the supernatant was cleared by filtering through a 0.45-µm filter. The clarified supernatant was loaded onto a His TALON™ super-flow cartridge (Clontech) equilibrated with 10 ml of equilibration buffer (1× PBS, 300 mM NaCl, 1% Triton X-100) and the cartridge was washed with 10 ml of equilibration buffer and 20 ml of wash buffer (1× PBS, 300 mM NaCl, 2 mM imidazole, 1% Triton X-100). Bac-PrP was then eluted in 5 ml of elution buffer containing complete protease inhibitor cocktail (EDTA-free) (Roche Diagnostics, Mannheim, Germany).

Partial purification of PrP^C^ from Tga20 transgenic mice brains was conducted as follows. Ten ml of 10% brain homogenate in 1× PBS, 1% SDC, 1% Triton X-100 and protease inhibitor cocktail (Nacalai Tesque) was incubated on ice with occasional agitation. After 2 min of sonication, the homogenate was centrifuged at 10,000× *g* for 10 min at 4°C. After filtering through a 0.45-µm filter, the supernatant was loaded onto a His TALON™ super flow cartridge (Clontech). The cartridge was equilibrated, washed and eluted as described above.

### Proteinase K and heat treatment of cell lysate

Five hundred microliters of HighFive™ cell suspension (5×10^4^/µl in DW; Invitrogen, Carlsbad, CA), SF21 cell suspension (5×10^4^/µl in DW; Invitrogen), N2a cell suspension (1.25 mg/µl in DW) and *Escherichia coli* DH5α suspension (1.25 mg/µl in DW) were mixed with 500 µl of 2× PMCA buffer (2× PBS, 8 mM EDTA, 2% Triton X-100) respectively. Proteinase K (PK) was added to the mixtures to a final concentration of 100 µg/ml. Following sonication for 1 min, the cell lysate was incubated at 37°C for 1 h. After incubation, the cell lysate was heated at 100°C for 2.5 h to inactivate PK. The resultant cell lysates were designated as PKHF, PKSF21, PKN2a and PK *E. coli*, respectively.

### Preparation of brain homogenate and PK treatment

To avoid contamination, the *Prnp^0/0^* mouse brain homogenate (*Prnp^0/0^* BH) was prepared in a laboratory in which infected materials had never been handled. Brains of *Prnp^0/0^* mice were homogenized at a 20% (w/v) concentration in 1× PBS containing a complete protease inhibitor cocktail (Roche). The BH was stored at −80°C until further use. The BH was mixed with an equal volume of 2× PMCA buffer, and the 10% (w/v) BH was subjected to PMCA. The mouse-adapted scrapie strains, Chandler, 79A, 22L, ME7, Obihiro [27], Tsukuba-2 (unpublished data) and mouse-adapted typical BSE (mBSE) were used as PrP^Sc^ seeds. These prion strains were propagated in ICR mice. The brains of mice at the terminal stage of the disease were homogenized at 10% concentration (w/v) in 1× PBS.

### PMCA

PMCA was carried out using the automatic cross-ultrasonic protein activating apparatus (ELESTEIN 070-GOT, Elekon Science Corp., Chiba, Japan), as reported previously (27). Amplification was performed with 40 cycles of sonication (pulse oscillation for 3 s repeated five times at intervals of 0.1 s), followed by incubation at 37°C for 30 min with gentle agitation. For PMCA using PK- and heat-treated cell lysates, the reaction mixture was prepared by adding 5 µl of IMAC-purified Bac-PrP (approximately 101 ng/µl) or 6 µl of IMAC-purified brain-derived PrP^C^ (approximately 125 ng/µl) to 10 µl of cell lysate and 85 µl of 1× PBS with 4 mM EDTA. For PMCA using whole insect cells expressing Bac-PrP, the PrP^C^ substrate was prepared by adding 5 µl of Bac-PrP virus-infected cell suspension (5×10^4^ cells/µl, approximately 110 ng/µl) to 95 µl of 10% *Prnp^0/0^* BH. For conventional PMCA using normal BH as the PrP^C^ substrate (BH-PMCA), 100 µl of 10% normal ICR mouse BH was used. The amplified products obtained after the first round of amplification were diluted 1∶10 with each PrP^C^ substrate and a second round of amplification was performed. This process was repeated when necessary.

### Western blotting

The PMCA products were digested with 50 µg/ml PK at 37°C for 1 h. An equal volume of 2× SDS sample buffer was added to the samples and boiled for 5 min. The samples were separated by SDS-PAGE in NuPAGE 12% Bis-Tris gels (Invitrogen), and transferred onto PVDF membranes. The membranes were probed with anti-PrP horseradish peroxidase conjugate monoclonal antibody T2 [28]. The blotted membrane was developed with SuperSignal West Dura Extended Duration Substrate (Pierce, Rockford, IL), and chemiluminescence signals were detected using Chemiimager (Alpha InnoTec, San Leandro, CA).

### Nuclease treatment of PKHF

Ribonuclease A (NipponGene) was added to PKHF at a final concentration of 500 µg/ml. DNase I (Takara) and benzonase (Novagen) were also added with 2 mM MgCl_2_ at final concentrations of 500 and 1000 units/ml, respectively. Following the addition of nuclease, the PKHFs were incubated at 37°C overnight with shaking (850 rpm) and then heat-treated at 100°C for 10 min to inactivate the nucleases.

### PCR

To obtain double stranded DNA fragments of different lengths, PCR was performed using Blend Taq polymerase (Toyobo, Tokyo, Japan). The thermocycling profile used for all products was 2 min at 94°C, followed by 40 cycles of 20 s at 94°C, 20 s at 55°C and 1 min at 72°C, followed by a final extension for 2 min at 72°C. The PCR products were purified using a QIAquick PCR purification kit (Qiagen, Valencia, CA, USA). The primers and templates used for the amplification of each product are as follows: for the 100-bp fragment derived from mouse GAPDH gene (M32599.1), 5′-GTATGACTCCACTCACGGCAAA-3′ and 5′-GGTCTCGCTCCTGGAAGATG-3′, mouse brain-derived cDNA. For 255-bp fragment from mouse PrP gene, 5′-AGCTGTCATATGGGCCAAGGAGGGGGTACCCATAATC-3′ and 5′-AGATGTGGATCCTCATCACCTGTAGTACACTTGGCA-3′, pCI-neo containing full-length mouse PrP. For 456-bp fragment from mouse PrP gene, 5′-AGCTGTCATATGGGC CAAGGAGGGGGTACCCATAATC-3′ and 5′-AGCTGTGGATCCTCATCAGGATCTTCTCCCGTCGTAATAG-3′, pCI-neo containing full-length mouse PrP. For 822-bp fragment from neomycin resistant gene, 5′-ATCCCCGGGGCCACCATGATTGAACAAG-3′ and 5′-GATGGTACCTCATCAGAAGAACTCGTCAAG-3′, pCI-neo (Promega, Madison, WI).

### Extraction of total lipids from brain

Brain total lipid was extracted from 0.1 g (equivalent to a brain) of freeze-dried *Prnp^0/0^* mouse brain powder twice using 6 ml of chloroform/methanol (2∶1, v/v) and 7.2 ml of chloroform/methanol (1∶2∶0.8, v/v/v). The extract was suspended in 200 µl of water, and 5 µl of the suspension were added to 100 µl of 1× PBS, 0.2% Triton X100, 4 mM EDTA and the PMCA reaction was performed.

### Bioassay

The PMCA products subjected to bioassay were prepared as follows. Chandler-infected BH was diluted 1∶1,000 with PrP^C^ substrate, and then one round of PMCA was performed. Next, the process of 10-fold dilution of the PMCA product and its subsequent amplification was repeated three times. After that, the process of five-fold dilution of the PMCA product and its subsequent amplification was repeated six times. For mBSE-seeded PMCA products, infected BH was diluted 1∶1,000, and then one round of PMCA was performed. Next, the process of 10-fold dilution of the PMCA product and its subsequent amplification was repeated 12 times. For the negative control, normal BH was diluted 1∶1,000, and then 13 rounds of sequential amplification was performed in a manner similar to that of mBSE amplification. The PMCA products were diluted 1∶10 with 1× PBS, and 20 µl of each diluted sample was injected intracerebrally into 3-week-old C57BL/6J mice under sevoflurane anesthesia. The mice were housed in a biosafety level 3 room of our animal facility and their clinical status was monitored at least three times per week. Animals (total 21 mice) were euthanized by sevoflurane overdose following evidence of progressive neurologic dysfunction or more than 421 days post inoculation for control, and the brains were removed. The right hemisphere of the brain was fixed in formalin for histopathology analysis and the left hemisphere was stored at −80°C for biochemical analysis. The average survival times of the experimental groups were analyzed by one-way ANOVA and the Tukey-Kramer multiple comparison test.

### Histopathological studies

The right hemisphere was fixed in 10% buffered formalin solution. Coronal slices of the brain were cut and immersed in 98% formic acid to reduce infectivity, and then embedded in paraffin wax. Sections with a thickness of 4 µm were cut and stained with hematoxylin and eosin (HE), or analyzed by immunohistochemistry. For the neuropathological analysis, the lesion profile was determined from the HE-stained sections by scoring the vacuolar changes in nine standard gray matter areas, as described previously [29]. For immunohistochemistry, PrP^Sc^ was detected in brain sections by the hydrated autoclaving method using anti-PrP monoclonal antibody 31C6 against the epitope of amino acids 143–149 of the mouse prion protein [30]. Immunoreactions were developed using anti-mouse universal immuno-peroxidase polymer (Nichirei Histofine Simple Stain MAX-PO (M), Nichirei, Tokyo, Japan) as the secondary antibody, and 3-3′ diaminobenzedine tetrachloride as the chromogen.

## Results

### Cofactor activity of insect cell lysates became functional after proteinase K digestion and heat treatment

**Figure 1:**
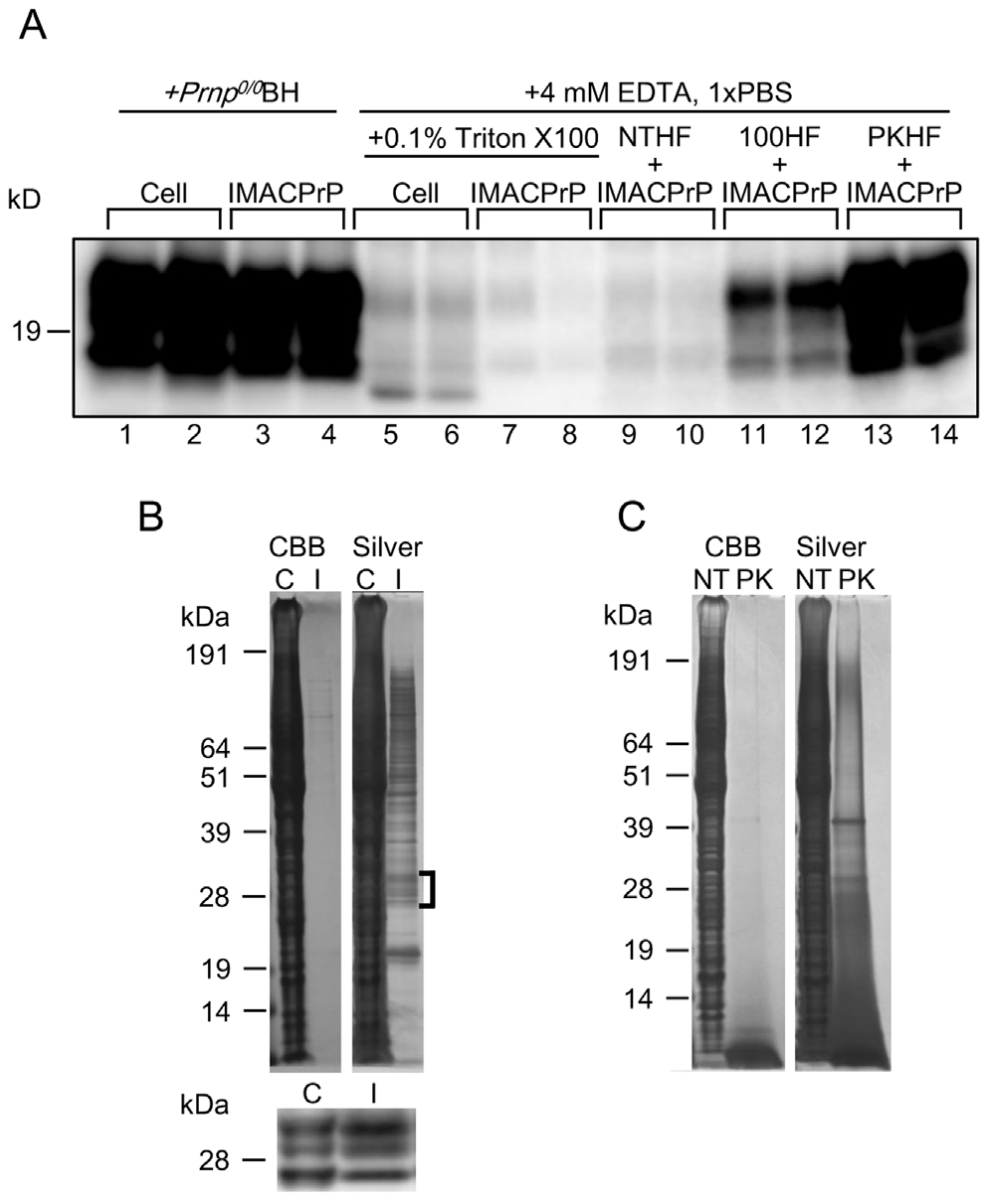
Amplification of PK-resistant Bac-PrP by PMCA. (A) Bac-PrP expressing cells (Cell) or IMAC-purified Bac-PrP (IMACPrP) were used as PrP^C^ sources, and Chandler PrP^Sc^ (diluted 1∶1000) was amplified in the presence of 10% *Prnp^0/0^* brain homogenate (lanes 1–4; *Prnp^0/0^*BH) or without the brain homogenate (lanes 5–8; 0.1% Triton X100). IMAC-purified Bac-PrP was also used as substrate in PMCA in the presence of non-treated HF cell lysate (NTHF), heat-treated HF cell lysate (100HF) or PK- and heat-treated HF cell lysate (PKHF) (lanes 9–14). All samples in lanes 9–14 contained 0.1% Triton X100, 4 mM EDTA, 1× PBS. All amplifications were carried out in duplicate. The PMCA products were digested with PK (50 µg/ml) at 37°C for 1 h, and analyzed by Western blotting. (B) Bac-PrP expressing cells (C; approximately 110 ng of Bac-PrP in 5×10^4^ cells) and IMAC-purified Bac-PrP fraction (I; approximately 101 ng of Bac-PrP and 1050 ng of concomitant proteins) were separated by SDS-PAGE and the bands were visualized by Coomassie Brilliant Blue (CBB; left panel) or silver (right panel) staining. The square bracket indicates the positions of the bands derived from Bac-PrP. The lower panel shows the result of Western blot analysis of Bac-PrP expressing insect cells and IMAC-purified Bac-PrP near the marker with 28 kDa. (C) Non-treated HF cell lysate (NT; 5×10^4^ cells) and PK- and heat-treated HF lysate (PK; 5×10^4^ cells) were separated by SDS-PAGE and the bands were visualized by CBB (left panel) or silver (right panel) staining.

As reported previously, proteinase K (PK)-resistant Bac-PrP (Bac-PrP^res^) was generated by PMCA using Bac-PrP expressing insect cells and *Prnp^0/0^* mouse brain homogenate (*Prnp^0/0^*BH) (KO-PMCA; Figure 1A, lanes 1 and 2). Bac-PrP was partially purified by about 37-fold from the lysates of Bac-PrP-expressing cells using immobilized metal ion affinity chromatography (IMAC). The proportion of Bac-PrP in the IMAC-purified fraction was approximately 8.8% of the total protein (Figure 1B, upper panel). Using partially purified Bac-PrP almost equivalent to that of Bac-PrP expressing cells (Figure 1B, lower panel), Bac-PrP^res^ was effectively generated after amplification as observed in the amplification using Bac-PrP expressing cells (Figure 1A, lanes 3 and 4). In the absence of *Prnp^0/0^*BH, generation of Bac-PrP^res^ was not observed in PMCA using Bac-PrP-expressing insect cells or IMAC-purified Bac-PrP alone (Figure 1A, lanes 5–8).

Further, we examined insect cell lysates in terms of potential cofactor activity. HighFive™ (HF) cell lysate (10 µl of 2.5×10^4^ cells/µl in 1× PBS, 4 mM EDTA, 1% Triton X-100; NTHF) was added to the IMAC-purified Bac-PrP, and then amplified. As expected, no significant cofactor activity was detected in the cell lysate (Figure 1A, lanes 9 and 10). It is noteworthy that a significant cofactor activity became obviously observed in the cell lysates after protease digestion and heat treatment (PKHF) (Figure 1A, lanes 13 and 14). We designated this PMCA using PKHF and IMAC-purified Bac-PrP as PKHF-PMCA. Proteins in the HF cell lysate were digested with PK, and the samples were incubated at 100°C for 2.5 h to inactivate protease activity completely. Coomassie Brilliant Blue (CBB) staining of the gel loaded with PKHF indicated that almost all proteins in PKHF were digested with PK. However, several bands or smeared signals that might be peptide fragments or their aggregates were recognized by silver staining (Figure 1C). Although the heat treatment alone induced cofactor activity in the insect cell lysate (Figure 1A, 100HF, lanes 11 and 12), the signal intensity of Bac-PrP^res^ was significantly less than that of the amplification using protease-treated lysates. These results suggested that cofactors necessary for *in vitro* amplification of Bac-PrP^res^ were also present in the insect cells, but unlike mammalian cofactors, protease digestion and/or heat-induced alteration were required for the functional expression of insect-derived cofactors.

### Cofactor activity of insect and mammal cell lines for Bac-PrP^res^ amplification

**Figure 2:**
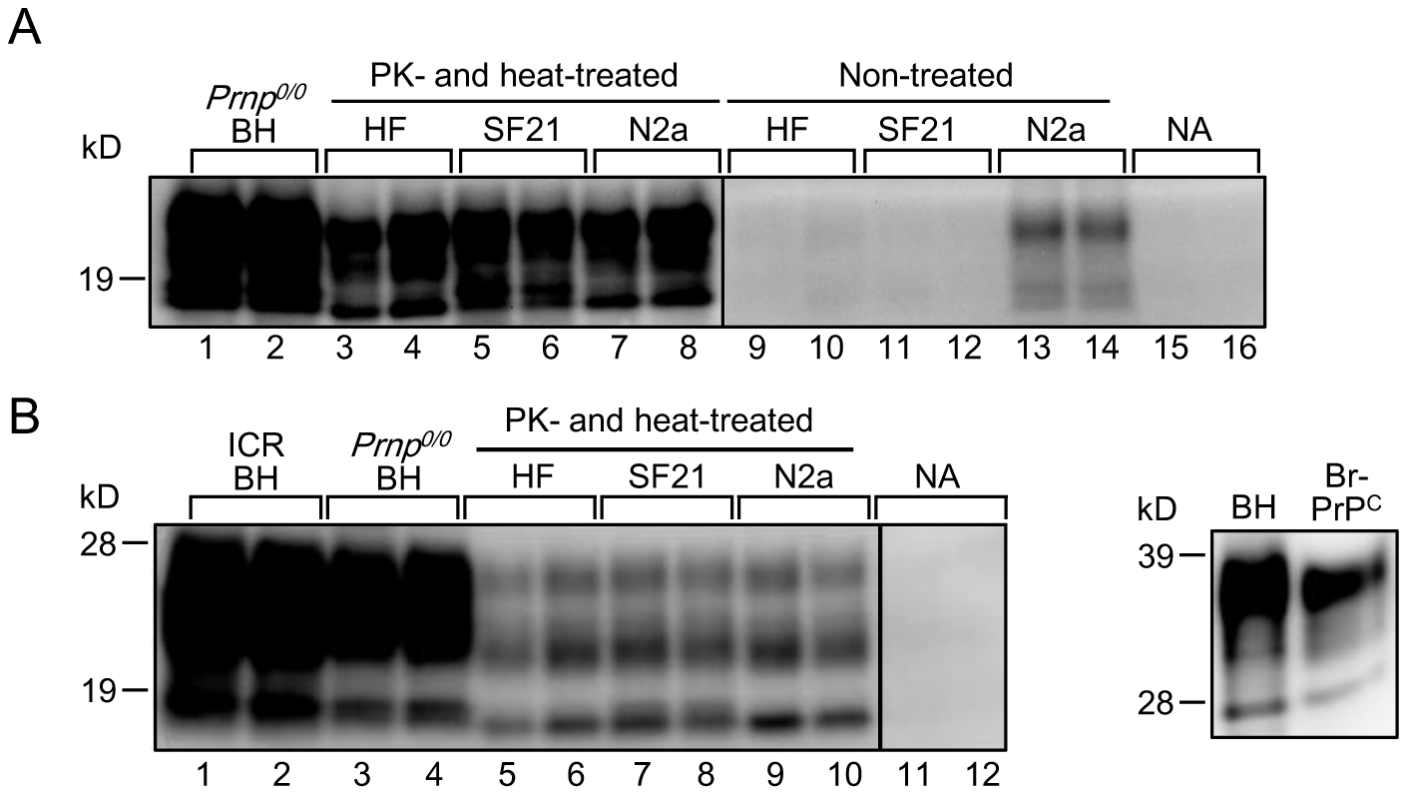
Cofactor activities of various cell lysates for *in vitro* conversion of Bac-PrP and brain-derived PrP^C^. (A) IMAC-purified Bac-PrP was used as the PrP^C^ source, and Chandler PrP^Sc^ (diluted 1∶1000) was amplified in the presence of *Prnp^0/0^*BH, PK- and heat-treated HF, SF21 and N2a lysates. Non-treated cell lysates (HF, SF21 and N2a) were also used for comparison of amplification. The PMCA products were digested with PK (50 µg/ml) at 37°C for 1 h, and analyzed by Western blotting. NA indicates the result of amplification without additives (IMAC-purified Bac-PrP alone). (B) IMAC-purified PrP^C^ derived from Tga20 mouse brains (Br-PrP^C^) was used as the PrP^C^ source, and Chandler PrP^Sc^ (diluted 1∶1000) was amplified in the presence of *Prnp^0/0^*BH, PK- and heat-treated HF, SF21, N2a or *Escherichia coli* lysates. The PMCA products were digested with PK (50 µg/ml) at 37°C for 1 h, and analyzed by Western blotting. NA indicates the result of amplification without any additives (IMAC-purified Br-PrP^C^ alone). Normal mouse BH (BH) was also used as a PrP^C^ source for comparison of amplification. The amount of IMAC-purified Br-PrP^C^ (approximately 750 ng/100 µl reaction solution) used as the PrP^C^ source was almost equal to that of the PrP^C^ contained in the 10% normal mouse BH (right panel). The western blot images are composite photos comprising band images from different gels.

To investigate whether other insect or mammal cell lines also possess cofactor activity for Bac-PrP^res^ amplification, we carried out PMCA using an insect cell line SF21 and a mouse neuroblastoma cell line N2a. Non-treated SF21 cell lysate did not induce Bac-PrP^res^ amplification as observed in the HF cells (Figure 2A, lanes 11 and 12). However, N2a cell lysate induced a low level of amplification of Bac-PrP^res^ without PK and heat treatments (Figure 2A, lanes 13 and 14). PK and heat treatments of the SF21 cell lysate induced a significant cofactor activity for Bac-PrP^res^ amplification; the cofactor activity of N2a cell lysate was also enhanced by these treatments (Figure 2A, lanes 3–8). These results indicated that the expression of cofactor activity upon PK and heat treatments was not specific to the HF cell line, and these treatments were also effective for the insect and mammalian cell lines examined. In contrast to the insect cells, at least partial cofactor activity was recognized in the N2a cell lysate without PK and heat treatments.

Next, we examined the cofactor activity of PK-and heat-treated insect cell lysates using brain-derived PrP^C^ as the substrate. Mammalian PrP^C^ was purified from Tga20 mouse brain homogenates by IMAC (Figure 2B right panel). Amplification of Chandler-derived mammalian PrP^res^ was observed using PK- and heat-treated HF, SF21 and N2a cell lysates (Figure 2B, lanes 5–10), although the amplification activity was lower than *Prnp^0/0^* BH. These results showed that the PK- and heat-treated cell lysates can also enhance the amplification of mammalian PrP^C^ to PrP^res^.

### RNA and DNA stimulate the amplification of Bac-PrP^res^ in PKHF-PMCA

**Figure 3:**
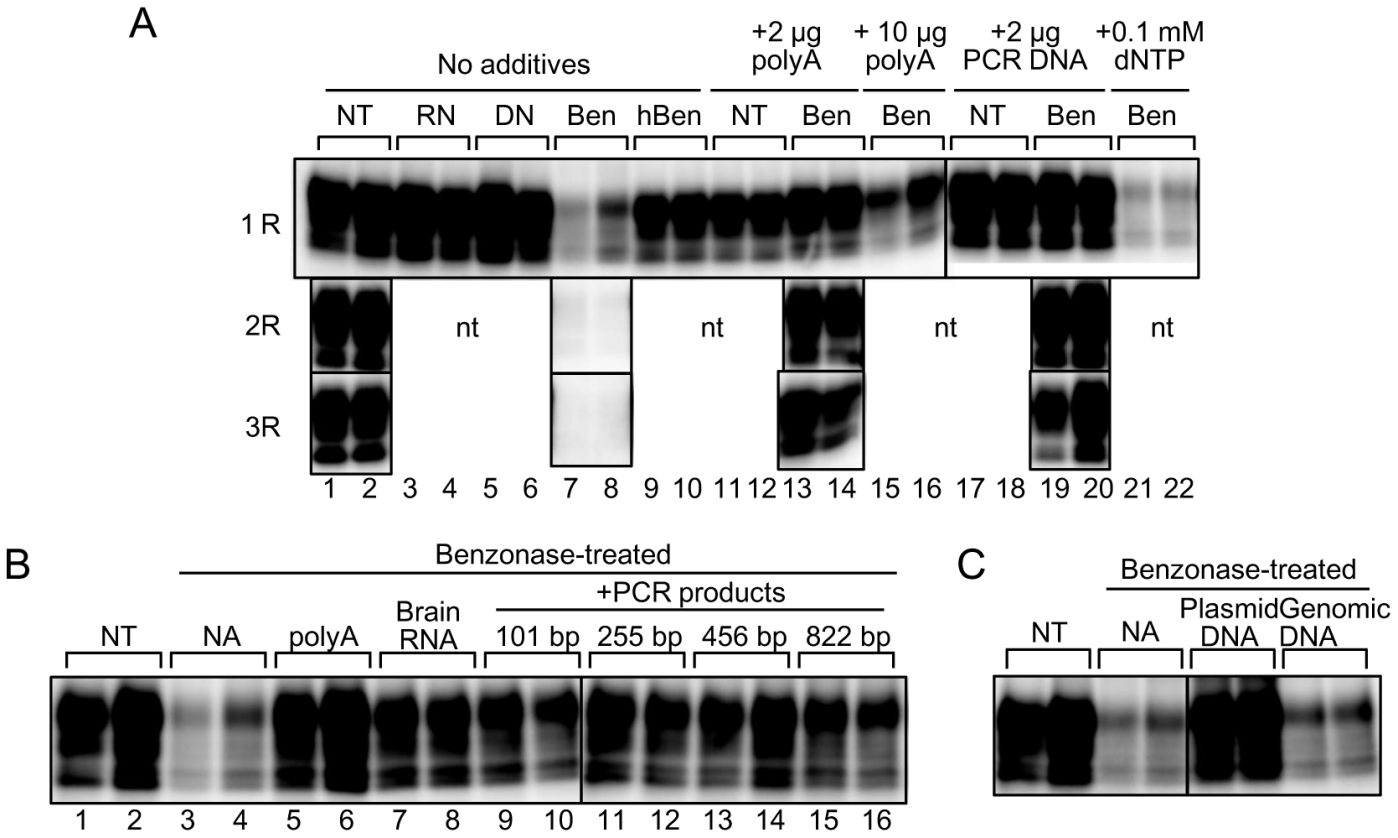
Cofactor activities of nucleic acids for Bac-PrP^res^ amplification. (A) PMCA was performed in the presence of non-treated PKHF (NT), PKHF digested with RNase- (RN), DNase- (DN), benzonase- (Ben) or heat-inactivated benzonase (hBen). PMCA using benzonase-treated PKHF was performed also in the presence of synthetic polyA (2 or 10 µg), DNA fragment (2 µg of purified 822-bp PCR product) or dNTP mixture (final 0.1 mM). Three rounds of amplification were performed using non-treated and benzonase-treated PKHF with and without polyA or PCR product. NT indicates not tested. Chandler PrP^Sc^ (diluted 1∶1000) was used as seed. (B) PMCA was performed using PKHF (NT) or benzonase-treated PKHF. PMCA using benzonase-treated PKHF was conducted also in the presence of polyA (2 µg), brain-derived total RNA (2 µg) or PCR products (2 µg) of 101 bp (mouse GAPDH gene), 255 bp (mouse prion protein gene), 456 bp (mouse prion protein gene) and 822 bp (neomycin resistance gene). Chandler PrP^Sc^ (diluted 1∶1000) was used as seed. (C) PMCA was conducted in the presence of PKHF (NT) or benzonase-treated PKHF. PMCA using benzonase-treated PKHF was performed also in the presence of pVL 1393 plasmid DNA (2 µg; 9639 bp; AB Vector, San Diego, CA) and genomic DNA (2 µg) isolated from the muscles of a goat. Chandler PrP^Sc^ (diluted 1∶1000) was used as seed. The western blot images are composite photos comprising band images from different gels but same membranes.

As demonstrated above, the cofactors for replication of Bac-PrP^res^ are suggested to be non-proteinaceous biomolecules shared by both mammals and insects. Several reports have shown that RNA facilitated PrP^Sc^ amplification in PMCA. Therefore, we investigated whether nucleic acids are involved in the process of replication of Bac-PrP^res^ in PKHF-PMCA. Nucleic acids were degraded and decreased after PK and heat treatments, but fragments ranging from about 100 to 1000 bp were obviously detected in PKHF (Figure S1A, lanes 1 and 2). Nucleic acids remained in RNase- or DNase-treated PKHF, although they were degraded to undetectable levels in benzonase-treated PKHF (Figure S1A, lanes 3–5). The amplification efficiencies of Bac-PrP^res^ in Chandler-seeded PKHF-PMCA were unaffected by RNase- or DNase-treatment (Figure 3A, lanes 1–6). However, benzonase treatment decreased the signal intensity of Bac-PrP^res^ by approximately 70% (Figure 3A, lanes 7 and 8), and Bac-PrP^res^ was lost during serial amplification (Figure 3A, lanes 7 and 8, 2R and 3R). In contrast, heat-inactivated benzonase treatment did not affect Bac-PrP^res^ amplification (Figure 3A, lanes 9 and 10). Next, we investigated whether the Bac-PrP^res^ amplification activities of benzonase-treated PKHF can be restored by the addition of nucleic acids such as synthetic polyadenylic acid (polyA), brain-derived total RNA, and PCR products. The addition of polyA recovered the amplification activities of benzonase-treated PKHF, equivalent to that of untreated PKHF (Figure 3A, lanes 13–16), and enabled serial amplification of Bac-PrP^res^ (Figure 3A, lanes13 and 14, R2 and R3). Total RNA from mouse brains also showed effective rescue capability (Figure 3B, lanes 7 and 8). The size of the polyA ranged from 200 bp to 6 kb, and the total RNA contained mainly 18S (1.8 kb) and 28S ribosomal RNA (4.8 kb) (Figure S1B, lanes 1 and 2). The addition of PCR products of 101–822 bp in length (Figure S1B, lanes 3–6) also recovered the amplification activities of benzonase-treated PKHF (Figure 3A, lanes 19 and 20; Figure 3B, lanes 9–16) Meanwhile, the addition of dNTP did not affect the amplification efficiency of Bac-PrP^Sc^ (Figure 3A, lanes 21 and 22). In addition to the PCR products, circular plasmid DNA (approximately 9.6 kb) effectively recovered the amplification activity of benzonase-treated PKHF, but the larger genomic DNA failed to rescue amplification activity (Figure 3C). These results suggested that in addition to RNA, DNA fragments of appropriate sizes (101 to 9600 bp) also acted as cofactors for Bac-PrP^res^ amplification, and their nucleotide sequences and structures (linear or circular) had no marked influence on cofactor activity.

### Effect of lipids on amplification of Bac-PrP^res^

**Figure 4:**
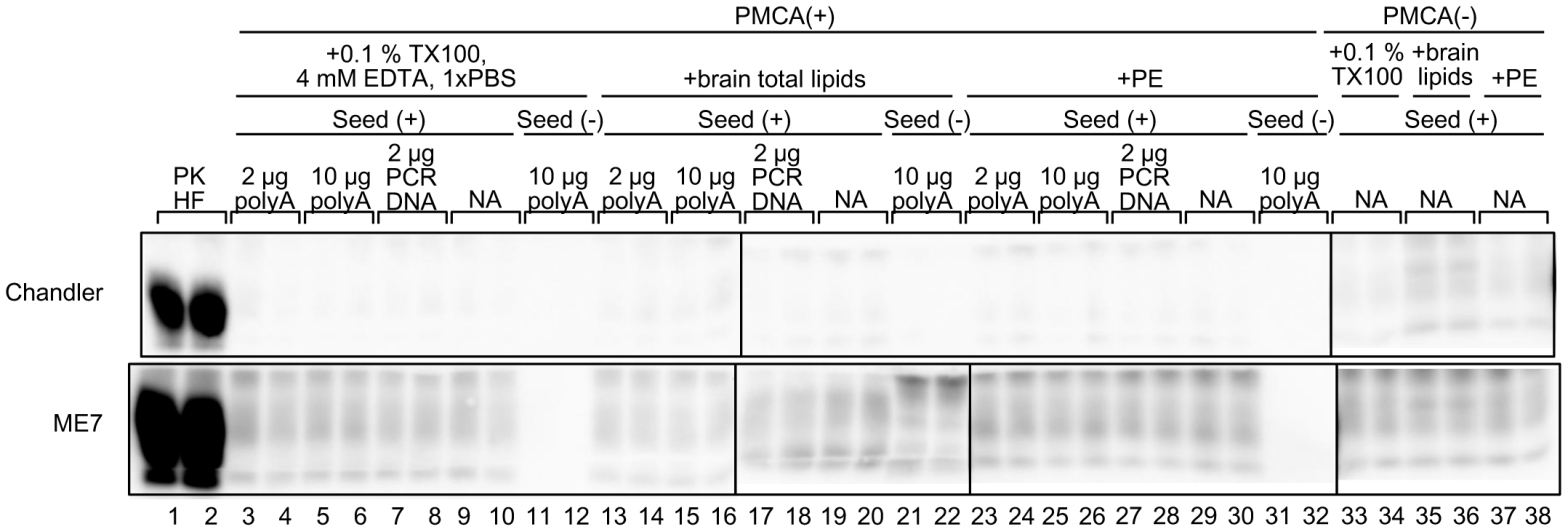
Effect of lipids on Bac-PrP^res^ amplification. IMAC-purified Bac-PrP was used as the PrP^C^ source, and Chandler and ME7 PrP^Sc^ (diluted 1∶1000) was amplified in buffer solution (0.1% Tx100, 4 mM EDTA, 1× PBS) in the absence (NA) or presence of nucleic acids (polyA, 2 or 10 µg; purified 822-bp PCR product, 2 µg). PMCA was also performed in buffer solution containing brain-derived total lipids (equivalent to 2.5 mg of brains) or synthetic phosphatidylethanolamine (18∶1, P1223, Sigma, 2.5 mM). Amplification using PKHF was performed for comparison. NA indicates no additives. Each sample was digested with PK (50 µg/ml) at 37°C for 1 h, and analyzed by Western blotting. Western blot analysis was also performed prior to amplification to determine the signal intensity of each PrP^Sc^ seed (lanes 33–36). The western blot images are composite photos comprising band images from different gels but same membranes.

Although Bac-PrP^res^ replication by PKHF-PMCA using Chandler PrP^Sc^ seed was largely attributable to the cofactor activities of RNA and DNA with particular sizes, benzonase-treatment did not completely inhibit the cofactor activity of PKHF. This observation suggested that other factors were also involved in Bac-PrP^res^ amplification in the PKHF-PMCA. Recently, it has been reported that synthetic phosphatidylglycerol and RNA or synthetic phosphatidylethanolamine (PE) alone facilitate the conversion of purified *E. coli*-derived recombinant PrP (*E. coli*-PrP) into PrP^Sc^ [6], [31]. We therefore investigated the cofactor activity of lipids for the amplification of Bac-PrP^res^ under the condition of PBS buffer containing TritonX-100 and EDTA. The signal intensity of PrP^res^ did not differ very much before and after the amplification, indicating that total lipids extracted from mouse brains (Figure 4, lanes 13–20) and synthetic PE (Figure 4, lanes 23–30) induced no significant PrP^res^ amplification with either strain in the presence or absence of nucleic acids such as polyA or DNA. These observations suggested that synthetic PE was not a functional cofactor for the amplification of Bac-PrP^res^ in the present experimental condition. Moreover, the total lipids present in the mouse brains showed little effect on the amplification of Bac-PrP^res^. Another implication of the experimental results was that nucleic acids alone were not effective for Bac-PrP^res^ amplification. These results suggested that other cooperative substances were necessary for the expression of the cofactor activity of nucleic acids.

### Cofactor activities of PKHF and *Prnp^0/0^* BH for various prion strains

**Figure 5:**
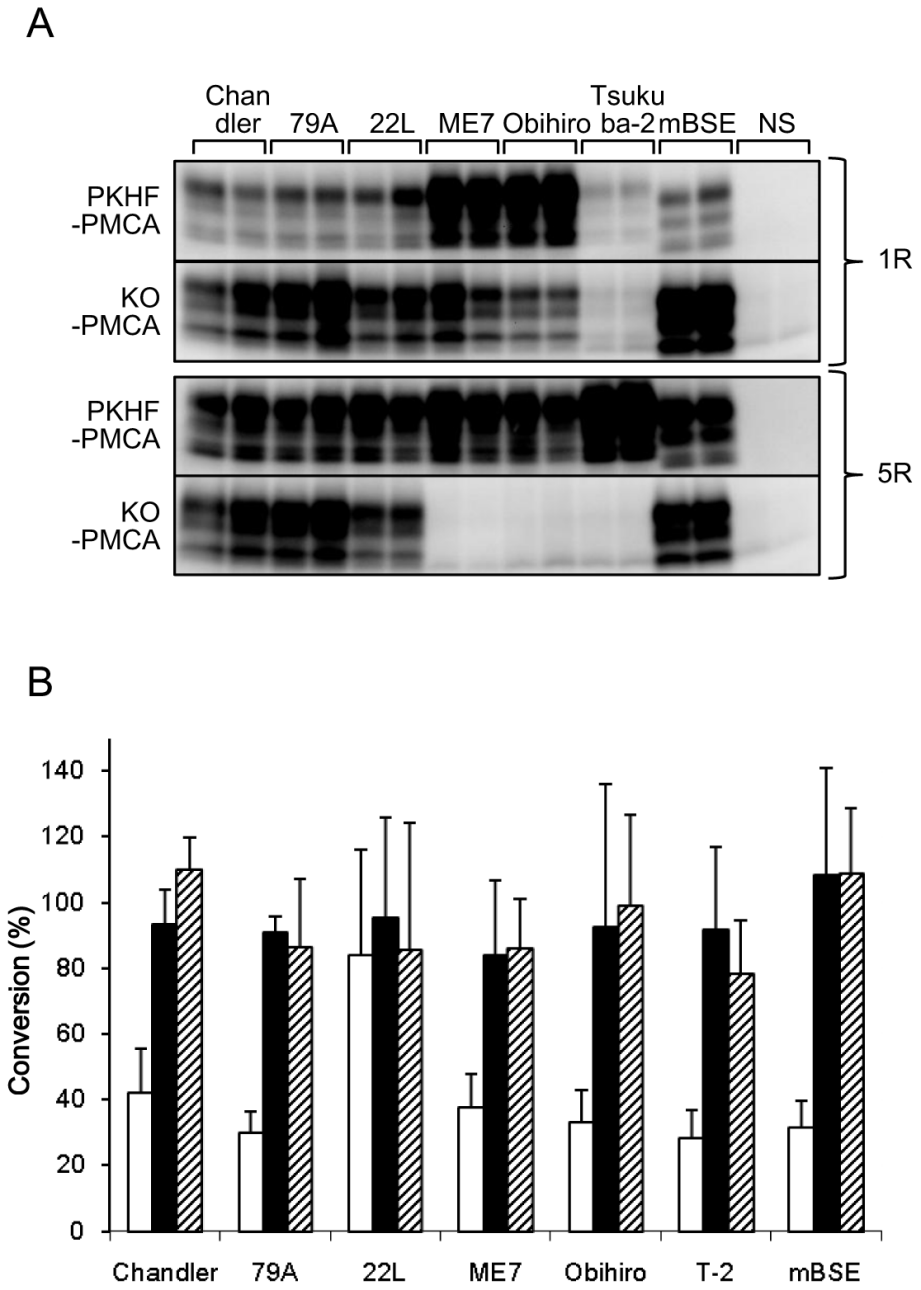
PKHF cofactor preference of prion strains. (A) PrP^Sc^ (diluted 1∶1000) from seven mouse-adapted prion strains was serially amplified using IMAC-purified Bac-PrP and PKHF (PKHF-PMCA) or Bac-PrP expressing cells and *Prnp^0/0^*BH (KO-PMCA). The PMCA products were digested with PK (50 µg/ml) at 37°C for 1 h and analyzed by Western blotting. The results of the first and fifth rounds of amplification are presented; profiles from the second to fourth rounds of amplification of each strain are provided in Figure S2. NS indicates the no-seed control. The Western blot images in panel A are composite photos comprising band images from different gels. (B) IMAC-purified Bac-PrP was used as the PrP^C^ source and PrP^Sc^ (diluted 1∶1000) from six scrapie and one BSE mouse-adapted prion strains were amplified in the presence of non-treated PKHF or benzonase-treated PKHF. For PMCA using benzonase-treated PKHF, amplification was performed in the absence (white bars) or presence of synthetic polyA (2 µg; black bars) or plasmid DNA (2 µg; hatched bars). The conversion efficiencies for each sample were expressed as a percentage change (mean ± standard error (SE) relative to the control value (control = 100)). The conversion value of PMCA using non-treated PKHF was used as a control. The results were analyzed by one-way ANOVA and the Tukey-Kramer multiple comparison test. PMCA was performed between three to six times.

Our results revealed that nucleic acids contained in PKHF were essential cofactors for the amplification of Chandler Bac-PrP^Sc^. To determine whether nucleic acids are also effective for the amplification of Bac- PrP^Sc^ of other prion strains, we used the PrP^Sc^ from six mouse-adapted scrapie strains and one BSE strain (mBSE) as seeds for PMCA in the presence or absence of nucleic acids. All PrP^Sc^ samples examined were amplified by one round of PKHF-PMCA in the presence of nucleic acids (Figure 5A, top panel). Following the digestion of nucleic acids with benzonase, the cofactor activity of PKHF was reduced significantly in the amplification using Chandler, 79A, ME7, Obihiro, Tsukuba-2, and mBSE PrP^Sc^ seeds (Figure 5B). A statistically significant improvement in amplification efficiency of Bac-PrP^Sc^ was observed in these prion strains after the addition of synthetic polyA to benzonase-treated PKHF relative to the additive-free cases. RNA-depleted plasmid DNA also showed an effect similar to polyA, and there were no significant differences between RNA and DNA in these strains. These results suggested that both RNA and DNA were involved in Bac-PrP^Sc^ amplification for these prion strains. With regard to 22L, PrP^Sc^ amplification efficiency was not significantly affected by benzonase treatment in the first round of amplification (Figure 5B), and PrP^Sc^ signal intensity remained to be low during four rounds of amplifications (Figure S2). On the other hand, 22L PrP^Sc^ signal intensity increased drastically after the third round of amplification using non-treated PKHF (Figure S2 and Figure 5B). Therefore, it is highly possible that nucleic acids act as a cofactor for the amplification of 22L PrP^Sc^.

Next, we compared the amplification efficiency of PrP^Sc^ between PKHF-PMCA and KO-PMCA (Figure 5B). Although insect cells expressing Bac-PrP were used as the PrP^C^ source for KO-PMCA, insect cell-derived cofactor activity was not involved in KO-PMCA because the insect cell-derived cofactors were not able to function without protease digestion and heat treatment. After one round of PKHF-PMCA, the signal intensities of Bac-PrP^res^ in the ME7 and Obihiro strains were higher than those of KO-PMCA. In the case of the amplification of Chandler, 79A, 22L, and mBSE PrP^Sc^ seeds, the amplification efficiencies of PKHF-PMCA were less than those of KO-PMCA. PKHF-PMCA and KO-PMCA had very low Bac-PrP^res^ generation rates in the Tsukuba-2 strain after one round of amplification. However, the Bac-PrP^res^ signal was easily detectable after five rounds of serial PKHF-PMCA in Tsukuba-2, and Bac-PrP^res^ signals were maintained or enhanced during five rounds of PKHF-PMCA in the other strains (Figures S2 and 5B). On the other hand, Bac-PrP^res^ signals were undetectable after five rounds of serial KO-PMCA in the ME7, Obihiro, and Tsukuba-2 strains. These results showed that PKHF-PMCA was more effective than KO-PMCA for continuous generation and replication of Bac-PrP^res^ in various prion strains suggesting that the cofactors involved in Bac-PrP^Sc^ amplification were different between PK and heat-treated insect cell lysates and brain homogenates.

### Detection sensitivity of PKHF-PMCA

**Figure 6:**
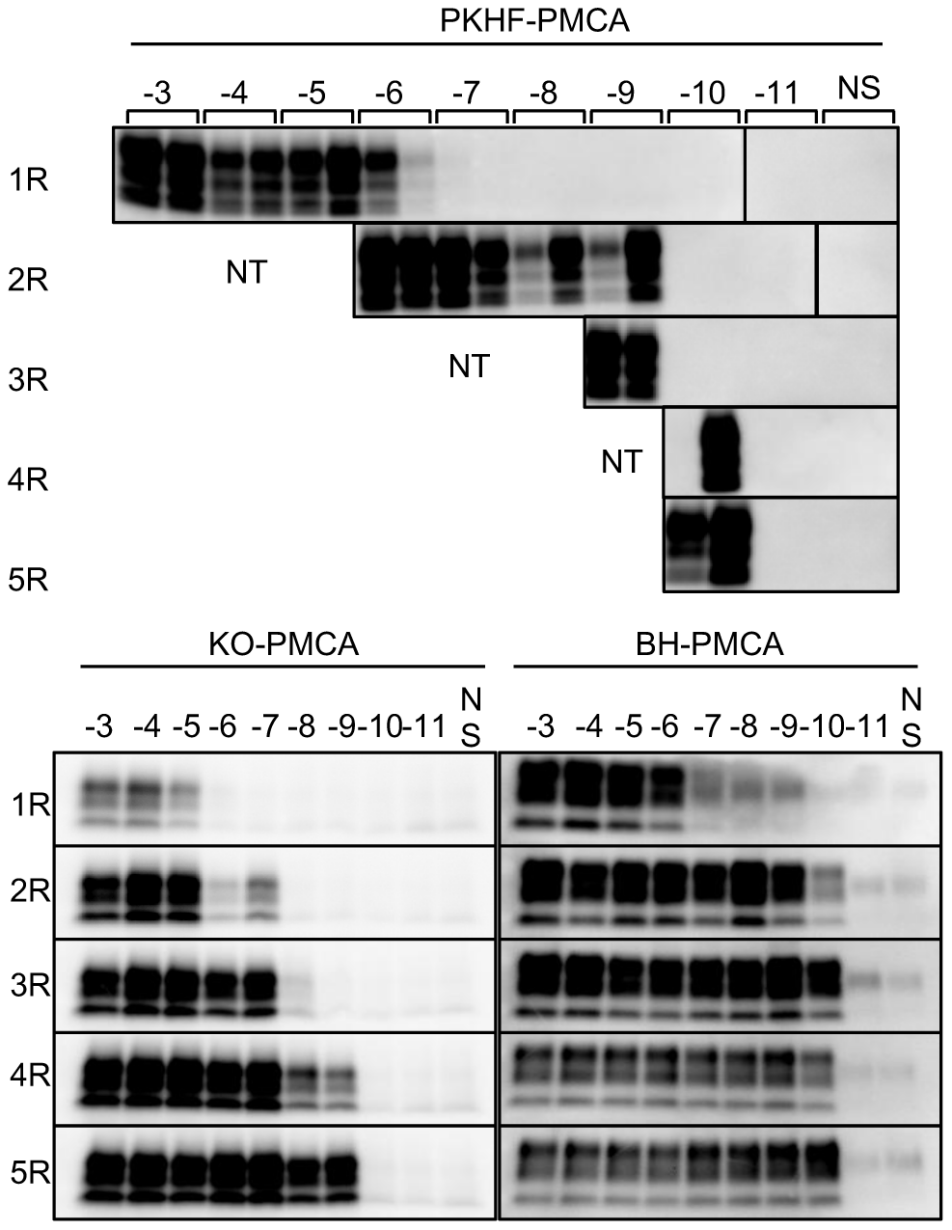
Detection sensitivity of PrP^Sc^ of a mouse-adapted BSE prion strain by PKHF-, KO- and BH-PMCA. mBSE-infected BH (10%) were diluted 10^−3^ to 10^−11^ and amplified by serial PMCA using IMAC-purified Bac-PrP and PKHF (PKHF-PMCA), Bac-PrP expressing cells and *Prnp^0/0^*BH (KO-PMCA) or normal brain BH (BH-PMCA). The PMCA products of each round (1R–5R) of amplification were digested with PK (50 µg/ml) at 37°C for 1 h, and analyzed by Western blotting. NS and NT indicate the no seed-control and not tested, respectively. The western blot images resulting from PKHF-PMCA are composite photos comprising band images from different gels.

We compared the amplification efficiency of PrP^Sc^ by PMCA using three different combinations: insect cofactors and insect PrP substrate (PKHF-PMCA), mammalian cofactors and insect PrP substrate (KO-PMCA), and mammalian cofactors and mammalian PrP substrate (BH-PMCA, Figure 6). In the case of the amplification of mBSE PrP^Sc^, the PMCA was highly sensitive and exhibited similar detection sensitivities for the PrP^Sc^ after four rounds of amplification: PrP^res^ could be detected up to 10^−10^ dilution by PKHF-PMCA and BH-PMCA, and up to 10^−9^ dilution by KO-PMCA. However, the amplification efficiencies were considerably different in the case of ME7 PrP^Sc^. KO-PMCA was less useful for the sequential amplification of ME7 PrP^Sc^, as shown in Figure 5. BH-PMCA was also insufficient to maintain ME7 PrP^Sc^ sequential amplification [23]. On the other hand, PrP^res^ could be amplified efficiently by serial PKHF-PMCA, and detected up to 10^−9^ dilution of ME7-infected BH after four rounds of amplification (Figure S3). These results suggested that PKHF-PMCA using insect cofactors worked well for ultrasensitive detection of PrP^Sc^ in these mouse-adapted prion strains, as compared to PMCA using mammalian cofactors.

### Biological and biochemical properties of PKHF-PMCA products

To investigate the infectivity of the PKHF-PMCA product, the amplified products obtained after 10 (Chandler-seeded) or 13 (mBSE-seeded) rounds of serial PMCA, which did not contain seed PrP^Sc^ were intracerebrally inoculated to C57BL/6J mice. The PMCA products were infectious, and the mice inoculated with the products obtained by serial amplification of Chandler and mBSE PrP^Sc^ developed the disease after 162±9 days (n = 8) and 193±11 days (n = 7), respectively. Control mice administered non-seeded PMCA products survived more than 421 days (n = 6).

**Figure 7:**
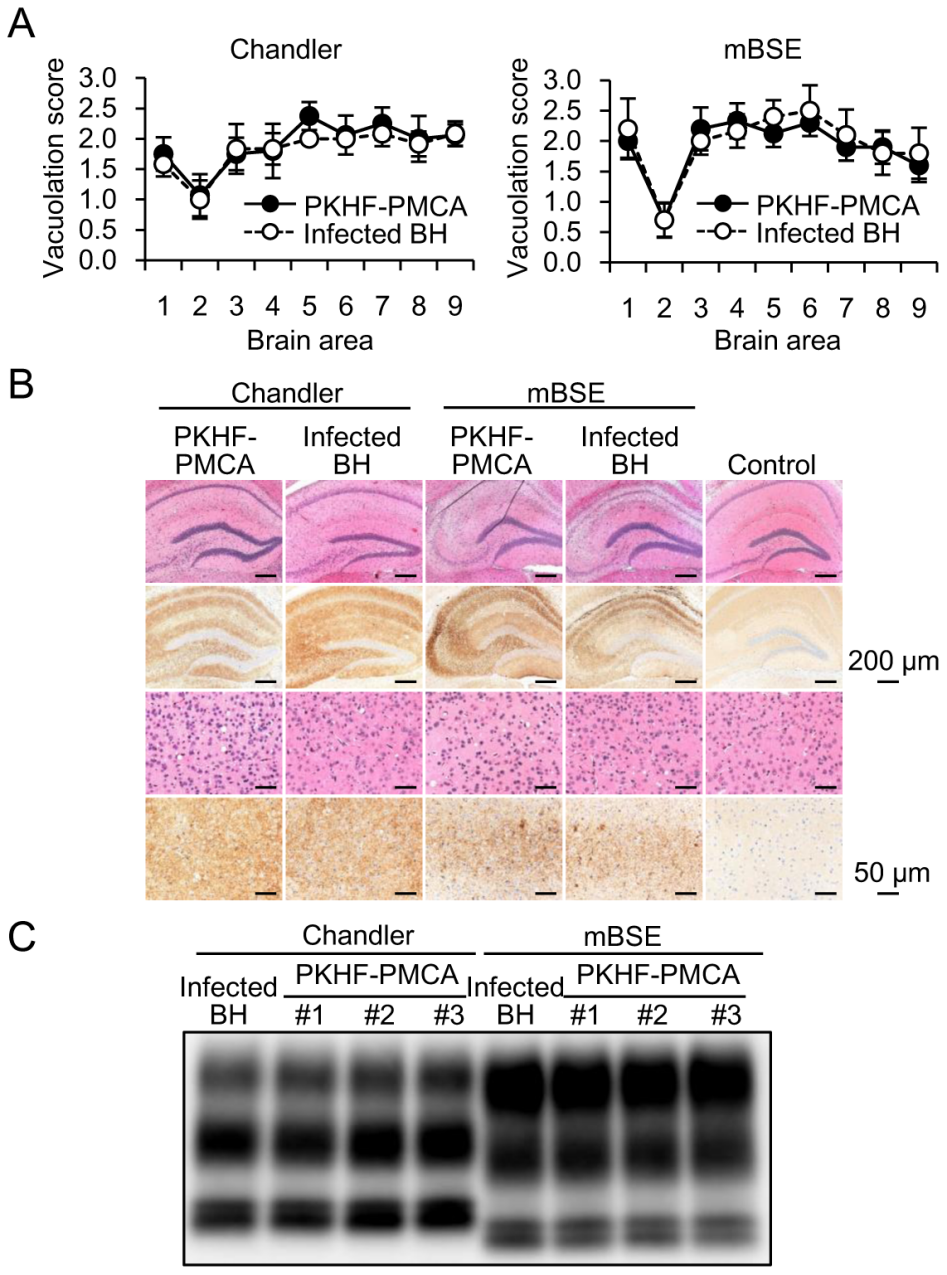
Histopathological and biochemical properties of PrP^Sc^ accumulated in brains of mice inoculated with PKHF-PMCA products. (A) Vacuolation profile in different brain areas of C57BL/6J mice inoculated with the PKHF-PMCA products (PKHF-PMCA) or PrP^Sc^ used as a seed (infected BH). Chandler and mBSE PrP^Sc^ were serially amplified by PKHF-PMCA, and the products obtained after 10 (Chandler) and 13 rounds (mBSE) of amplification were inoculated into the mice. Brain regions are as follows: 1, dorsal medulla; 2, cerebellar cortex; 3, superior collicullus; 4, hypothalamus; 5, thalamus; 6, hippocampus; 7, septal nuclei of the paraterminal body; 8, cerebral cortex at the level of 4 and 5; 9, the cerebral cortex at the level of 7. The average lesion scores (5 to 10 animals/group) and SE are shown. (B) Vacuolation (first and third rows) and PrP^Sc^ accumulation (second and fourth rows) in the brains inoculated with the PMCA products (PKHF-PMCA) and PrP^Sc^ used as the seed (infected BH). Mice inoculated with the products obtained after 13 rounds of unseeded PMCA were used as the control. (C) Western blot profiles of PrP^Sc^ accumulated in the brains of C57BL/6J mice inoculated with the PKHF-PMCA products and PrP^Sc^ used as the seed (infected BH). Brain homogenates were prepared from three PKHF-PMCA product-inoculated mice and one brain of PrP^Sc^-inoculated mouse (infected BH).

Further, we examined the neuropathological properties of the PKHF-PMCA products. The regional profiles of neuronal vacuolation scores in the brains of the mice inoculated with the PMCA-derived PrP^Sc^ were very similar to those of the mice inoculated with the brain-derived PrP^Sc^ in both Chandler and mBSE prion strains (Figure 7A). Immunohistochemical analysis of the hippocampus and the occipital cortex of the affected mice revealed strain-specific characteristics in the mice inoculated with the PKHF-PMCA-derived PrP^Sc^ or the brain-derived PrP^Sc^. In the case of the Chandler strain, PrP^Sc^ accumulated over the entire region of the brain sections with diffuse distribution and synaptic-like immunostaining, and the accumulation patterns of PrP^Sc^ were very similar between mice inoculated with the PMCA-derived PrP^Sc^ and the brain-derived Chandler PrP^Sc^. In contrast, in the mBSE strain, dense granular PrP^Sc^ deposition in the hippocampus and peculiar lamellar accumulation of PrP^Sc^ in the occipital cortex were observed in mice inoculated with the PMCA-derived PrP^Sc^ and brain-derived mBSE PrP^Sc^. Moderate degenerated neurons in the CA1 region of the hippocampus were found commonly in these mice.

In addition, the glycosylation patterns and molecular weights of PrP^Sc^ accumulated in the brains of the mice inoculated with PKHF-PMCA-derived PrP^Sc^ were indistinguishable from those of the mice inoculated with brain-derived PrP^Sc^ of the Chandler and mBSE strains (Figure 7C). These results suggested that the PKHF-PMCA using only insect cell-derived substances was capable of amplifying infectious PrP^Sc^ with strain-specific pathogenic properties.

## Discussion

In the current study, we demonstrated that insect cells also contained the cofactors required for *in vitro* replication of GPI-anchored Bac-PrP^Sc^ that preserved the strain characteristics of the PrP^Sc^ seed. Mammalian cofactors contaminating the PrP^Sc^ seeds appeared to not participate in the replication because purified PrP^Sc^ (SAF) was also amplified effectively by PKHF-PMCA (Figure S4). Unlike brain-derived mammalian cofactors, PK digestion and/or heat treatment were necessary for the cofactor activities in insect cell lysates. We demonstrated that both RNA and DNA were important for PrP^Sc^ amplification via reconstitution experiments following benzonase treatment, although it has been shown that DNA could be substituted for RNA in RML PrP^Sc^ amplification under RNA-depleted PMCA conditions [32]. Furthermore, we found that the nucleic acid dependency of *in vitro* amplification of Bac-PrP^Sc^ was not necessarily the same among prion strains.

There are several possible explanations for the lack of cofactor activity in the untreated insect-cell lysates. For example, most non-proteinaceous cofactors, such as nucleic acids, might exist in conjugated or aggregated states with cellular proteins in the insect-cell lysate; therefore, the active sites necessary for the expression of cofactor activities might be masked by such forms. Protease digestion or heat treatment of cellular proteins might allow the cofactors to become functional by dissociating the aggregates. Alternatively, the non-proteinaceous cofactors might be functional even in untreated cell lysates, but some kind of inhibitory factors present in the insect cells might interfere with the conversion of PrP^C^ to PrP^Sc^. The latter possibility is strongly supported by the results that amplification of Bac-PrP^Sc^ was efficiently inhibited by adding untreated insect cell lysates into the PKHF-PMCA reaction solution (Figure S5). Such inhibitory factors could be proteins because their inhibitory activities were lost by protease digestion or heat treatment. Lactoferrin, a multifunctional glycoprotein belonging to the transferrin family, has been reported to inhibit PrP^Sc^ replication in murine cell lines and PrP^Sc^ amplification by PMCA [33]. Although insect cells produce transferrins that have various biological functions, including roles in iron transport, anti-oxidative stress, and anti-infective activity [34], there was no evidence that lactoferrin-like molecules were present in the cell lysates. Nonetheless, our results (Figure S5) strongly suggested that certain inhibitory factors were present in the insect cell lysate. Such inhibitory factors might also be contained in a certain type of mammalian cell lines and therefore, PK/heat-treatment might be necessary to express full cofactor activity in N2a cells (Figure 2A).

The functional roles of nucleic acids as cofactors are not clear. Nucleic acids contained in PKHF were fragmented to sizes of 100–1000 bp, likely due to digestion with endogenous nucleases and denaturation by heat (Figure S1A). The size of the DNA significantly influenced cofactor activities, and DNA fragments or plasmids of 101–9600 bp functioned well as cofactors. Therefore, it was possible that the fragmentation of genomic DNA was involved in the expression of cofactor activity in insect cells. Fragmented and deproteinized nucleic acids could acquire the ability to interact with PrP to enhance the structural conversion of PrP^C^ to PrP^Sc^. Alternatively, the binding of PrP to nucleic acids of appropriate sizes could prevent random PrP aggregate formation, or such nucleic acids might serve as a scaffold for the orderly accumulation of PrP^Sc^.

In PKHF-PMCA, nucleic acid depletion had little effect on the first round of PrP^Sc^ amplification in the 22L strain (Figure 5A). This observation suggested that cofactors, other than nucleic acids, were involved in PKHF-PMCA. Synthetic phospholipids, such as phosphatidylglycerol (POPG) or phosphatidylethanolamine (PE), are known to act as cofactors for the generation and amplification of GPI-anchorless *E. coli*-PrP [6], [31]. However, in the presence of brain-derived total lipids or PE, and even with the combination of these lipids and nucleic acids, Bac-PrP^res^ was not generated by PMCA using Chandler and ME7 PrP^Sc^ (Figure 4). In addition to nucleic acids and phospholipids, polysaccharides, including glycosaminoglycans (GAG), sulfated dextran, and heparin, are known to stimulate PrP^res^ conversion [7], [35] or PrP^Sc^ amplification [16], [36]. Furthermore, low-molecular-weight molecules, such as Cu^2+^ ions [37], [38] and NADPH [11], have been suggested to be involved in the *in vitro* conversion of brain-derived PrP^C^ or *E. coli*-PrP. These PK-resistant and heat-stable molecules mentioned above may have been present in the lysate of insect cells and some factors in them could have either acted as a cofactor alone or participated cooperatively in the functional expression of the cofactor activity of nucleic acids. Further studies are needed to fully identify and understand the cofactors involved in the amplification of GPI-anchored Bac-PrP^Sc^, as well as to examine the cofactor preference of each prion strain, which may hold the key to the prion diversity problem. The reconstitution experiment using GPI-anchored Bac-PrP performed in the current study may be applied to the characterization of mammalian cofactors present in BH.

In conclusion, we revealed in this study that the essential cofactor molecules needed for the high-fidelity replication of mammalian PrP^Sc^ were also present in insect cells, where protease digestion and/or heat treatment were required for the functional expression of cofactor activity. We showed that DNA of appropriate sizes (101 to 9600 bp) represented some of the non-proteinaceous cofactor molecules in insect cells, but our results suggested that other cellular factors were necessary for amplification. Finally, PMCA using only insect cell-derived substances (iPMCA) resulted in successful amplification of various prion strains, showing low background (nonspecific) signals and high sensitivity.

## Supporting Information

Figure S1 **Images of nucleic acids from nucleases-digested PKHF and nucleic acids used for PMCA using benzonase-treated PKHF.** (A) Each 5 µl of non-treated HF cell lysate (NTHF), non-digested PKHF (ND), PKHF digested with RNase (R), DNase (D) or benzonase (B) was separated by agarose gel (1.5%) electrophoresis and the gel was stained with ethidium bromide. (B) PolyA (200 µg, lane 1) and mouse brain total RNA (2 µg, lane 2) were separated by agarose gel (1.2%) electrophoresis in the denatured condition. PCR products (200 ng) of 101 bp (lane 3), 255 bp (lane 4), 456 bp (lane 5) and 822 bp (lane 6) were separated by agarose gel (1.5%) electrophoresis. The gels were stained with ethidium bromide.
(TIF)

Figure S2 **Western blot analysis of the serial amplification of seven mouse-adapted prion strains.** IMAC-purified Bac-PrP was used as the PrP^C^ source, and each PrP^Sc^ (diluted 1∶1000) was serially amplified in the presence of PKHF (NT) or benzonase-treated PKHF (Ben). After one round of amplification (Fig. 5A), the PMCA products were subsequently amplified three times (2R–4R). Bac-PrP^res^ signals were maintained or enhanced during rounds 2R–4R of amplification in Chandler, 79A, ME7, Obihiro and mBSE strains. The signal intensity of Bac-PrP^res^ increased significantly after 3R in 22L. No obvious enhancement of the signal intensity was observed during 2R–4R in Tsukuba-2. For all prion strains examined, the amplification efficiency diminished by benzonase treatment. The western blot images are composite photos comprising band images from different gels.
(TIF)

Figure S3 **Detection sensitivity of ME7 PrP^Sc^ by serial PKHF-PMCA. IMAC-purified Bac-PrP was used as the PrP^C^ source, and ME7 PrP^Sc^ diluted 10^−3^ to 10^−11^ was serially amplified in the presence of PKHF.** NS and NT indicate no seed control and not tested, respectively. The western blot images are composite photos comprising band images from different gels.
(TIF)

Figure S4 **PKHF-PMCA using scrapie-associated fibril as the PrP^Sc^ seed.** (A) Scrapie-associated fibril (SAF) was prepared from Chandler and mBSE-infected mouse brains. The SAF samples (200 ng) were separated by SDS-PAGE and the bands were visualized by silver staining. (B) Chandler and mBSE PrP^Sc^ were amplified by PKHF-PMCA using infected-BH and the SAFs as seeds. In the case of Chandler, similar amounts of Bac-PrP^res^ were produced after amplification using infected BH (contained 4.8 ng of PrP^res^) or the SAF (10 ng). In contrast, mBSE-infected BH (contained 9.6 ng of PrP^res^) yielded a higher efficiency of amplification compared to mBSE SAF (10 ng). (C) The PKHF-PMCA products using SAF seeds were infectious, and all mice (*n* = 8) inoculated with the products developed prion disease (Chandler, 174±8 days; mBSE, 208±3 days). Western blot analysis indicated that the band patterns were very similar between the PrP^Sc^ in brains inoculated with SAF-based PMCA products and those inoculated with infected BH-based PMCA products.
(TIF)

Figure S5 **The inhibitory effect of non-treated insect HighFive™ cell lysates (NTHF) on Bac-PrP^Sc^ amplification.** Ten microliters of non-treated HighFive™ cell lysates (2.5×10^4^/µl in 1% Triton X100, 4 mM EDTA, 1× PBS; NTHF) was added into PKHF-PMCA reaction solution, and PMCA was conducted. Bac-PrP^Sc^ amplification was efficiently inhibited by addition of NTHF.
(TIF)
