# Supplementary figures and images for: Insect Cell-Derived Cofactors Become Fully Functional after Proteinase K and Heat Treatment for High-Fidelity Amplification of Glycosylphosphatidylinositol-Anchored Recombinant Scrapie and BSE Prion Proteins
Source: PLoS One. 2013 Dec 18;8(12):e82538. doi: 10.1371/journal.pone.0082538 (PMC3867355; doi:10.1371/journal.pone.0082538)

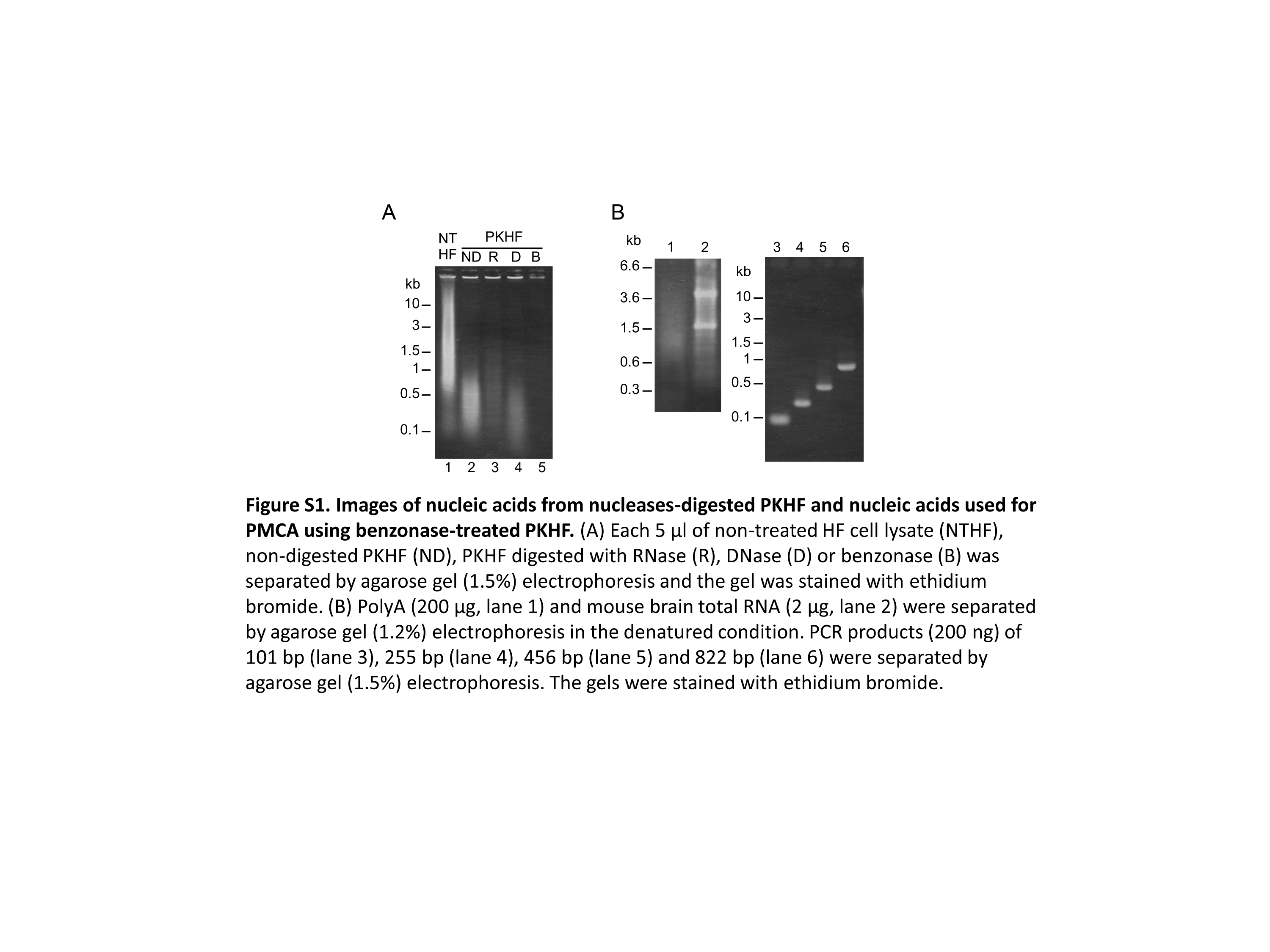

Supplement: Figure S1 — Images of nucleic acids from nucleases-digested PKHF and nucleic acids used for PMCA using benzonase-treated PKHF. (A) Each 5 µl of non-treated HF cell lysate (NTHF), non-digested PKHF (ND), PKHF digested with RNase (R), DNase (D) or benzonase (B) was separated by agarose gel (1.5%) electrophoresis and the gel was stained with ethidium bromide. (B) PolyA (200 µg, lane 1) and mouse brain total RNA (2 µg, lane 2) were separated by agarose gel (1.2%) electrophoresis in the denatured condition. PCR products (200 ng) of 101 bp (lane 3), 255 bp (lane 4), 456 bp (lane 5) and 822 bp (lane 6) were separated by agarose gel (1.5%) electrophoresis. The gels were stained with ethidium bromide. (TIF) [file pone.0082538.s001.tif]

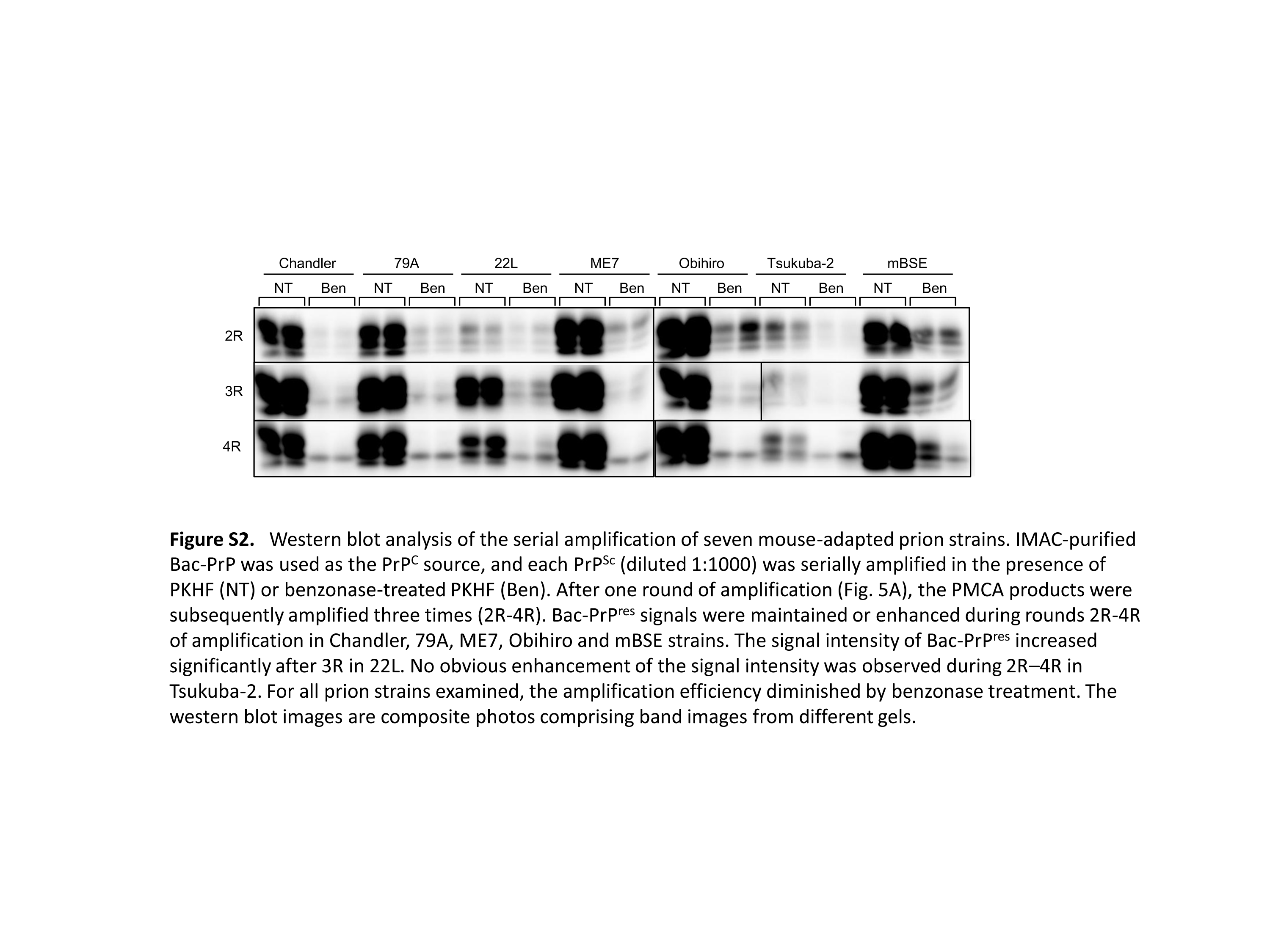

Supplement: Figure S2 — Western blot analysis of the serial amplification of seven mouse-adapted prion strains. IMAC-purified Bac-PrP was used as the PrPC source, and each PrPSc (diluted 1∶1000) was serially amplified in the presence of PKHF (NT) or benzonase-treated PKHF (Ben). After one round of amplification (Fig. 5A), the PMCA products were subsequently amplified three times (2R–4R). Bac-PrPres signals were maintained or enhanced during rounds 2R–4R of amplification in Chandler, 79A, ME7, Obihiro and mBSE strains. The signal intensity of Bac-PrPres increased significantly after 3R in 22L. No obvious enhancement of the signal intensity was observed during 2R–4R in Tsukuba-2. For all prion strains examined, the amplification efficiency diminished by benzonase treatment. The western blot images are composite photos comprising band images from different gels. (TIF) [file pone.0082538.s002.tif]

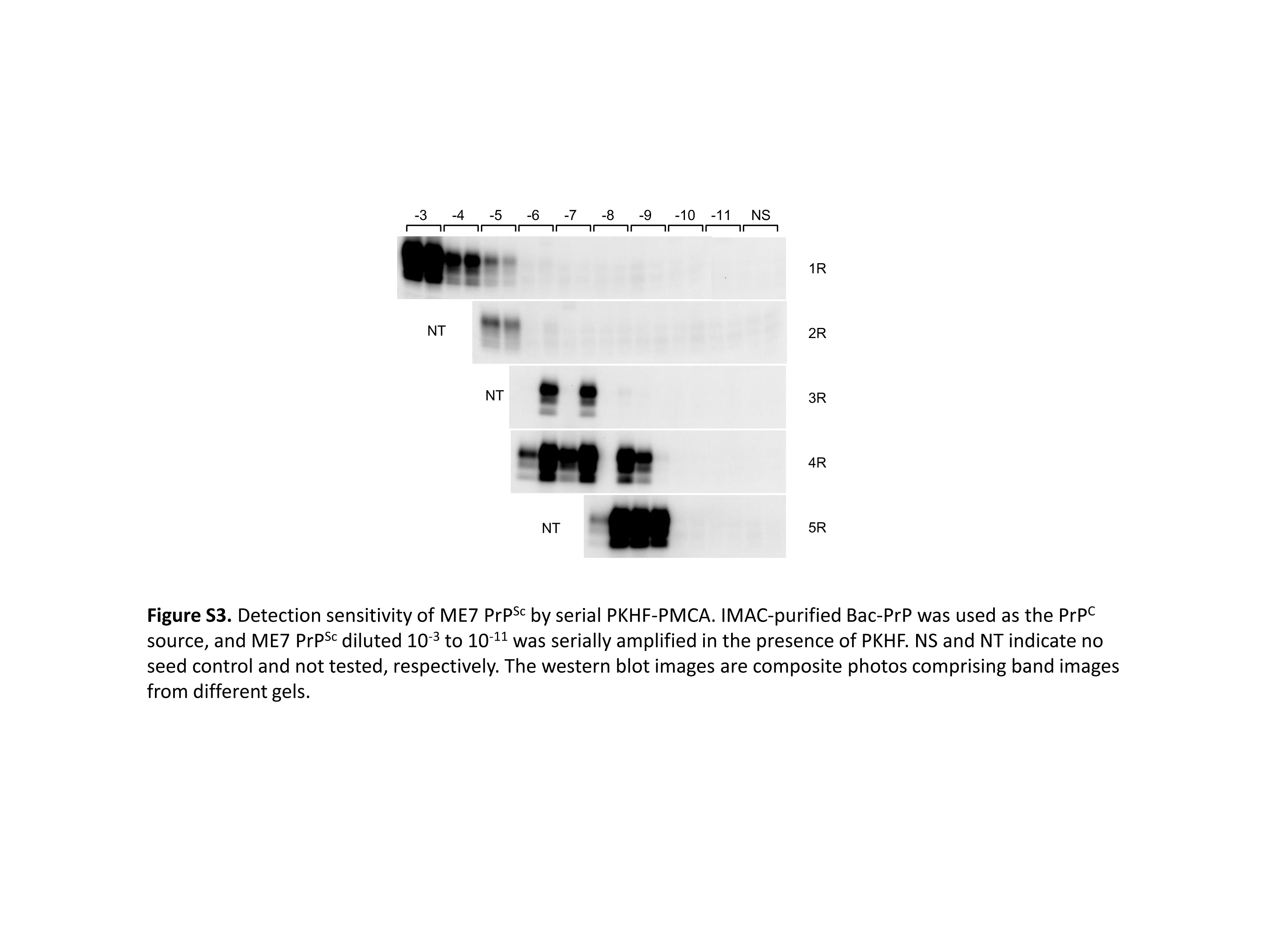

Supplement: Figure S3 — Detection sensitivity of ME7 PrPSc by serial PKHF-PMCA. IMAC-purified Bac-PrP was used as the PrPC source, and ME7 PrPSc diluted 10−3 to 10−11 was serially amplified in the presence of PKHF. NS and NT indicate no seed control and not tested, respectively. The western blot images are composite photos comprising band images from different gels. (TIF) [file pone.0082538.s003.tif]

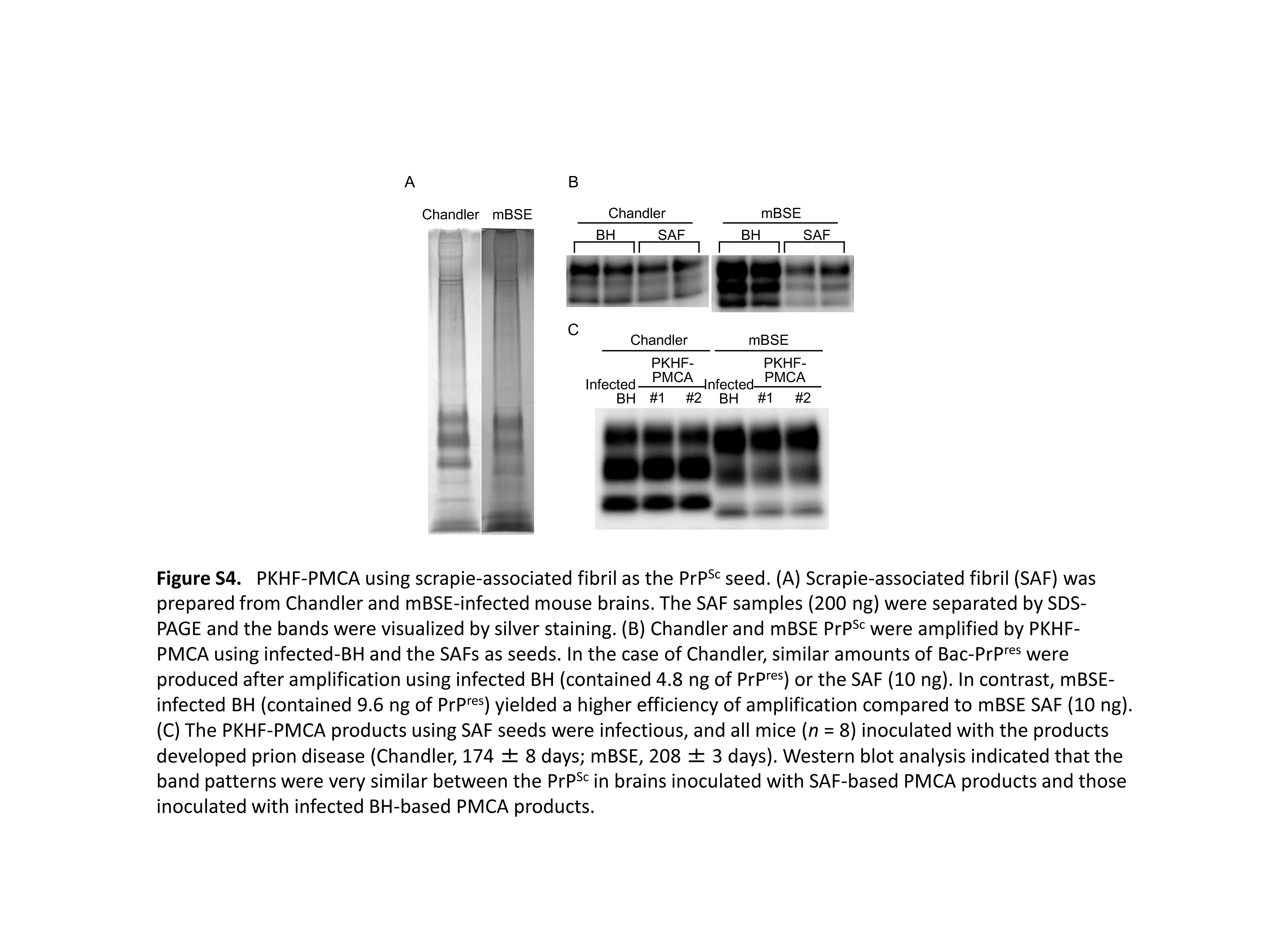

Supplement: Figure S4 — PKHF-PMCA using scrapie-associated fibril as the PrPSc seed. (A) Scrapie-associated fibril (SAF) was prepared from Chandler and mBSE-infected mouse brains. The SAF samples (200 ng) were separated by SDS-PAGE and the bands were visualized by silver staining. (B) Chandler and mBSE PrPSc were amplified by PKHF-PMCA using infected-BH and the SAFs as seeds. In the case of Chandler, similar amounts of Bac-PrPres were produced after amplification using infected BH (contained 4.8 ng of PrPres) or the SAF (10 ng). In contrast, mBSE-infected BH (contained 9.6 ng of PrPres) yielded a higher efficiency of amplification compared to mBSE SAF (10 ng). (C) The PKHF-PMCA products using SAF seeds were infectious, and all mice (n = 8) inoculated with the products developed prion disease (Chandler, 174±8 days; mBSE, 208±3 days). Western blot analysis indicated that the band patterns were very similar between the PrPSc in brains inoculated with SAF-based PMCA products and those inoculated with infected BH-based PMCA products. (TIF) [file pone.0082538.s004.tif]

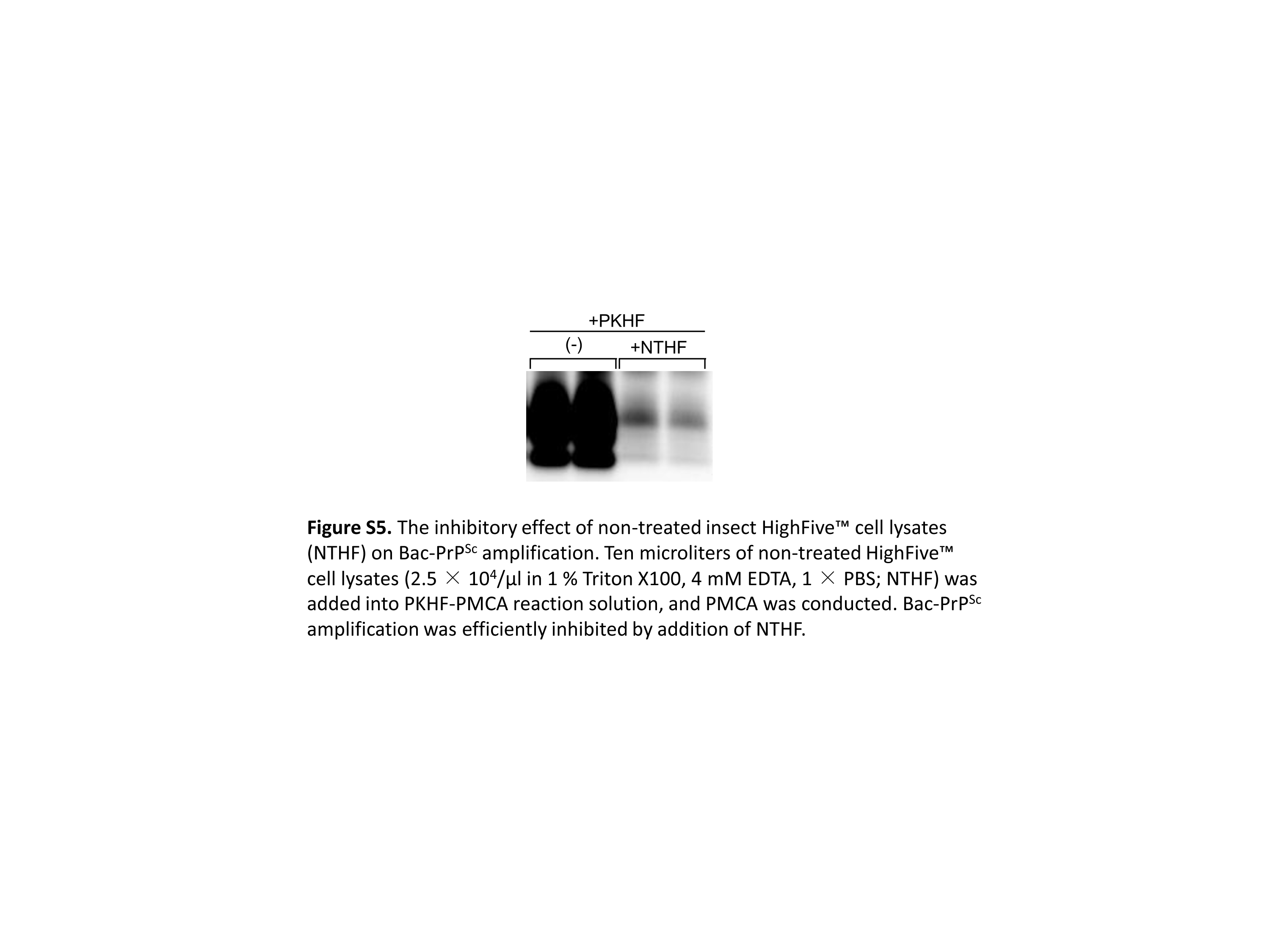

Supplement: Figure S5 — The inhibitory effect of non-treated insect HighFive™ cell lysates (NTHF) on Bac-PrPSc amplification. Ten microliters of non-treated HighFive™ cell lysates (2.5×10^4^/µl in 1% Triton X100, 4 mM EDTA, 1× PBS; NTHF) was added into PKHF-PMCA reaction solution, and PMCA was conducted. Bac-PrPSc amplification was efficiently inhibited by addition of NTHF. (TIF) [file pone.0082538.s005.tif]
